# Kaempferol and Chrysin Synergies to Improve Septic Mice Survival

**DOI:** 10.3390/molecules22010092

**Published:** 2017-01-06

**Authors:** Omar A. Harasstani, Chau Ling Tham, Daud A. Israf

**Affiliations:** Department of Biomedical Science, Faculty of Medicine and Health Sciences, Universiti Putra Malaysia, Serdang, 43400 Selangor, Malaysia; omar_harastany@yahoo.com (O.A.H.); chauling@upm.edu.my (C.L.T.)

**Keywords:** chrysin, kaempferol, polymicrobial sepsis

## Abstract

Previously, we reported the role of synergy between two flavonoids—namely, chrysin and kaempferol—in inhibiting the secretion of a few major proinflammatory mediators such as *tumor necrosis factor*-alpha (TNF-α), *prostaglandin E_2_ (PGE_2_)*, and nitric oxide (NO) from lipopolysaccharide (LPS)-induced RAW 264.7 cells. The present study aims to evaluate the effects of this combination on a murine model of polymicrobial sepsis induced by cecal ligation and puncture (CLP). Severe sepsis was induced in male ICR mice (*n* = 7) via the CLP procedure. The effects of chrysin and kaempferol combination treatment on septic mice were investigated using a 7-day survival study. The levels of key proinflammatory mediators and markers—such as *aspartate aminotransferase* (AST), TNF-α, and NO—in the sera samples of the septic mice were determined via ELISA and fluorescence determination at different time point intervals post-CLP challenge. Liver tissue samples from septic mice were harvested to measure myeloperoxidase (MPO) levels using a spectrophotometer. Moreover, intraperitoneal fluid (IPF) bacterial clearance and total leukocyte count were also assessed to detect any antibacterial effects exerted by chrysin and kaempferol, individually and in combination. Kaempferol treatment improved the survival rate of CLP-challenged mice by up to 16%. During this treatment, kaempferol expressed antibacterial, antiapoptotic and antioxidant activities through the attenuation of bacterial forming units, AST and NO levels, and increased polymorphonuclear leukocyte (PMN) count in the IPF. On the other hand, the chrysin treatment significantly reduced serum TNF-α levels. However, it failed to significantly improve the survival rate of the CLP-challenged mice. Subsequently, the kaempferol/chrysin combination treatment significantly improved the overall 7-day survival rate by 2-fold—up to 29%. Kaempferol and chrysin revealed some synergistic effects by acting individually upon multiple pathophysiological factors involved during sepsis. Although the kaempferol/chrysin combination did not exhibit significant antibacterial effects, it did exhibit anti-inflammatory and antioxidant activities, which translate to significant improvement in the survival rate of septic animals. These findings suggest the potential application of this combination treatment as a beneficial adjuvant supplement strategy in sepsis control.

## 1. Introduction

Sepsis is a serious life-threatening disorder that occurs as a response to invading organisms and their components. It is considered to be the leading cause of death in many intensive care units (ICUs) around the world, often with a high mortality rate, and remains without a definitive cure [1]. This complex syndrome is associated with the overproduction of inflammatory mediators [2], resulting in a systemic inflammatory response syndrome (SIRS) and, consequently, the development of multiple organ dysfunction syndrome (MODS) and tissue and organ fibrosis, which eventually lead to death [3,4]. The onset of the inflammatory response and septic shock progression is mainly mediated through a complex cascade of host responses, such as an increase in white blood cell population, nitric oxide (·NO), aspartate aminotransferase (AST), and tumor necrosis factor-alpha (TNF-α) levels in the patients’ sera [5,6]. Attenuation of these factors have been argued to be a possible strategy in sepsis control regimens [7].

Flavonoids are naturally occurring polyphenolic agents present in various plant sources, which express promising antioxidant, anti-inflammatory, and anticancer properties [8,9]. They have also been reported to possess multiple biological benefits, as reported in both in vivo and in vitro models. At the cellular level, flavonoids have long shown the ability to attenuate the production of major proinflammatory mediators from activated polymorphonuclear leukocytes (PMNs) [10]. Moreover, they have also been reported to improve the outcome of endotoxin shock in animal models [11]. Two flavonoids in particular, namely chrysin (5,7-dihydroxyflavone) and kaempferol (3,4′,5,7-tetrahydroxyflavone), from the flavone and flavonol family, respectively, have been reported to express multiple benefits in both animal and cellular models [12]. However, the possible beneficial outcomes of their combination on a sepsis model have not been previously investigated.

Recently, there has been a rising trend in pharmacological studies addressing the synergistic possibilities of multiple-agent combinations to achieve certain desirable therapeutic effects [13]. Ideally, a combination of treatments is considered synergistic when it expresses an increased therapeutic efficacy accompanied with a decreased dose. Based on our previous findings, the kaempferol/chrysin combination expressed promising synergistic effects by targeting multiple responses at the cellular level, including the attenuation of NO, prostaglandin E2 (PGE_2_), and TNF-α secretion from lipopolysaccharide (LPS)-induced RAW 264.7 cells [14].

·NO is a highly reactive free radical, which is produced in a number of tissues and exerts a wide range of physiological and pathophysiological effects [15]. During sepsis, the initial rise of ·NO levels has its beneficial effects, such as vasodilation and microcirculatory blood flow improvement. However, ·NO overproduction at later stages of sepsis may lead to several undesirable side effects, such as hypotension, impaired vasopressin secretion, and altered vascular permeability [16]. Accordingly, ·NO serves as a fundamental target in septic shock management [17,18].

Apart from that, sepsis development is associated with several other pathophysiological features, such as the sum increase of white blood cell population during sepsis progression [19] and the activation and recruitment of circulating PMNs to the site of injury following the release of key proinflammatory mediators [20]. Activated PMNs release myeloperoxidase (MPO) [21], which in turn provides an indirect means for assessing tissue injury by quantifying the amount of recruited PMNs in a tissue sample [22]. Likewise, prolonged sepsis leads to multiple organ fibrosis, and apoptotic myocytes and hepatocytes of the heart and liver release AST into the blood [23], which in turn is a useful serum fibrosis marker [24]: the greater the degree of tissue damage, the greater the degree of AST released, and decreasing levels of AST may indicate recovery [25]. Furthermore, TNF-α has been reported to play a major role in the early phase of sepsis [1,26], as it coordinates the immune system to delay human blood neutrophil apoptosis indirectly by upregulating proinflammatory cytokines such as interleukin-8 [27,28]. All together, these pathophysiological features serve as important clinical biomarkers to assess sepsis development and progression.

The cecal ligation and puncture (CLP) procedure has been widely used as a benchmark to induce polymicrobial sepsis in animal models. The infusion of Gram-negative and Gram-positive bacteria—among other components—from the punctured cecum by the CLP procedure mimics the natural course of sepsis seen in humans. This study investigates the possible synergistic outcomes of chrysin and kaempferol combinatorial treatment against CLP-induced septic shock in an animal model. This study also further investigates the effects of chrysin and kaempferol individually and in combination against several pathophysiological factors of sepsis—such as the anti-inflammatory, antioxidant, and antibacterial properties—during the progression of sepsis.

## 2. Results

### 2.1. Effects of Chrysin and Kaempferol Individual or Combination Treatments on Endotoxin Shock

**Figure 1:**
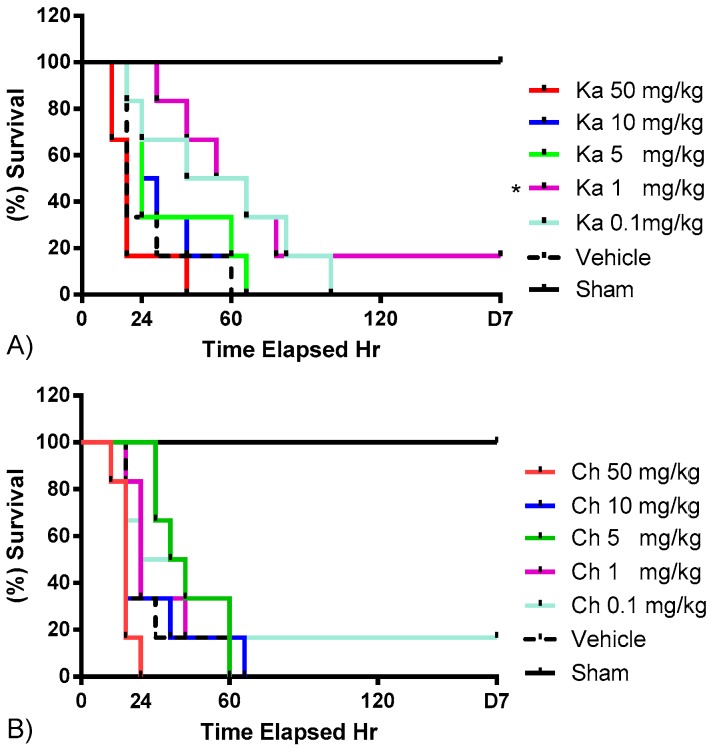
Kaplan–Meier plots of the survival rates (%) of cecal ligation and puncture (CLP)-challenged septic ICR mice treated with different doses of (**A**) kaempferol; (**B**) chrysin within 7 days. Animals were i.p. injected with treatments or vehicle (10 mL/kg) 1 h prior to CLP challenge, * *p* = 0.01.

**Figure 2:**
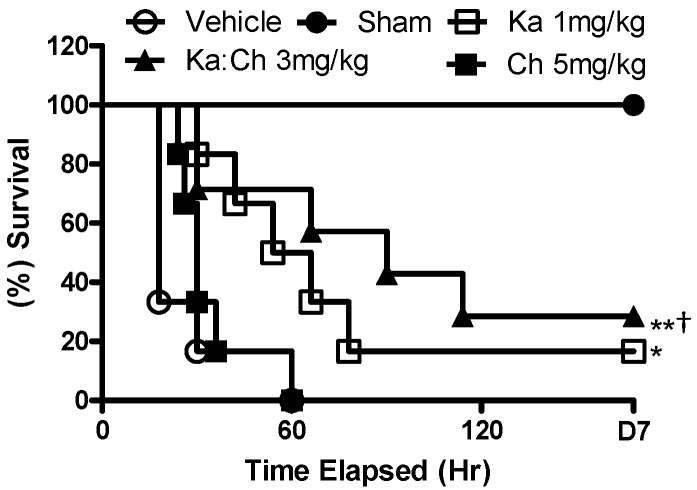
Effects of combinatorial treatments on the survival rate (%) of CLP-challenge septic mice within 7 days. CLP/Vehicle (2% DMSO and 5% Tween-20/phosphate-buffered saline (PBS)) (○); Sham (●); CLP/Ka:Ch 3 mg/kg (▲); CLP/Ka 1 mg/kg (□); CLP/Ch 5 mg/kg (■). Animals were i.p. injected with treatments or vehicle (10 mL/kg) 1 h prior to CLP challenge, * *p* ≤ 0.01 and ** *p* ≤ 0.005 versus CLP/vehicle, ^†^ *p* = 0.05 versus CLP/Ch chi-square and log-rank test).

The optimal treatment doses of chrysin and kaempferol were determined separately prior to formulating the kaempferol/chrysin combination. Figure 1 shows the survival dose–response of kaempferol and chrysin, respectively, for septic mice. Taking into account the highest survival rate and first mortality occurrence, chrysin at 5 mg/kg showed the best efficacy among other tested concentrations, whereas kaempferol at 1 mg/kg significantly reduced the mortality rate to 16% (*p* ≤ 0.05) compared to the CLP/vehicle control group. Consequently, a fixed-ratio, serial dilution dose–response study was constructed by combining the above two optimal doses (Figure 2). A kaempferol/chrysin combination at 3 mg/kg significantly increased survival rate up to 29% (*p* ≤ 0.01) compared to the CLP/vehicle control group. The sham-operated control group resulted in no mortality over the 7-day experiment, while all the mice from the CLP/vehicle-treated group died within the first 60 h.

### 2.2. Organ Weights

**Figure 3:**
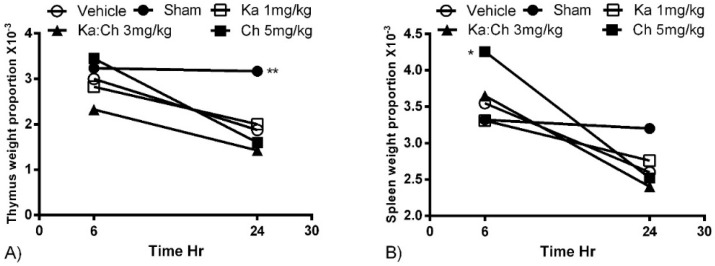
The effects of kaempferol and chrysin individual and combination treatments on mean organ weight proportions 6 and 24 h after CLP. Organs directly weighed after sacrificing the mice. (**A**) Thymus and (**B**) spleen. Each point represents the mean of four experiments. * *p* ≤ 0.05 and ** *p* ≤ 0.01 versus CLP/vehicle (Student’s *t*-test).

Chrysin treatment at 5 mg/kg transiently increased the mean spleen weight of the male ICR mice (*p* ≤ 0.05) at 6 h (Figure 3b). This was followed by a decreasing trend in the mean spleen weights of all groups to points below the sham-operated control group. Meanwhile, the mean thymus weights decreased during the progression of sepsis from 6 to 24 h, however, none of them were significant (Figure 3a).

### 2.3. Quantitative and Differential Leukocytic Count

**Figure 4:**
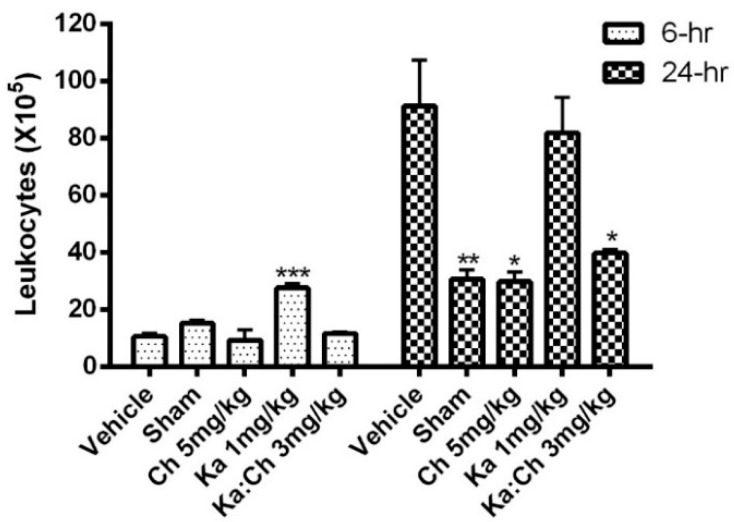
The effects of kaempferol and chrysin individual and combination treatments on intraperitoneal fluid (IPF) total white blood cell (WBC) count. Mice were i.p. injected with treatments or vehicle 1 h prior to CLP and sacrificed at 6 and 24 h time points. IPF was screened with trypan blue for total WBC count. * *p* ≤ 0.05, ** *p* ≤ 0.01, and *** *p* ≤ 0.001 versus CLP/vehicle (Student’s *t*-test).

**Figure 5:**
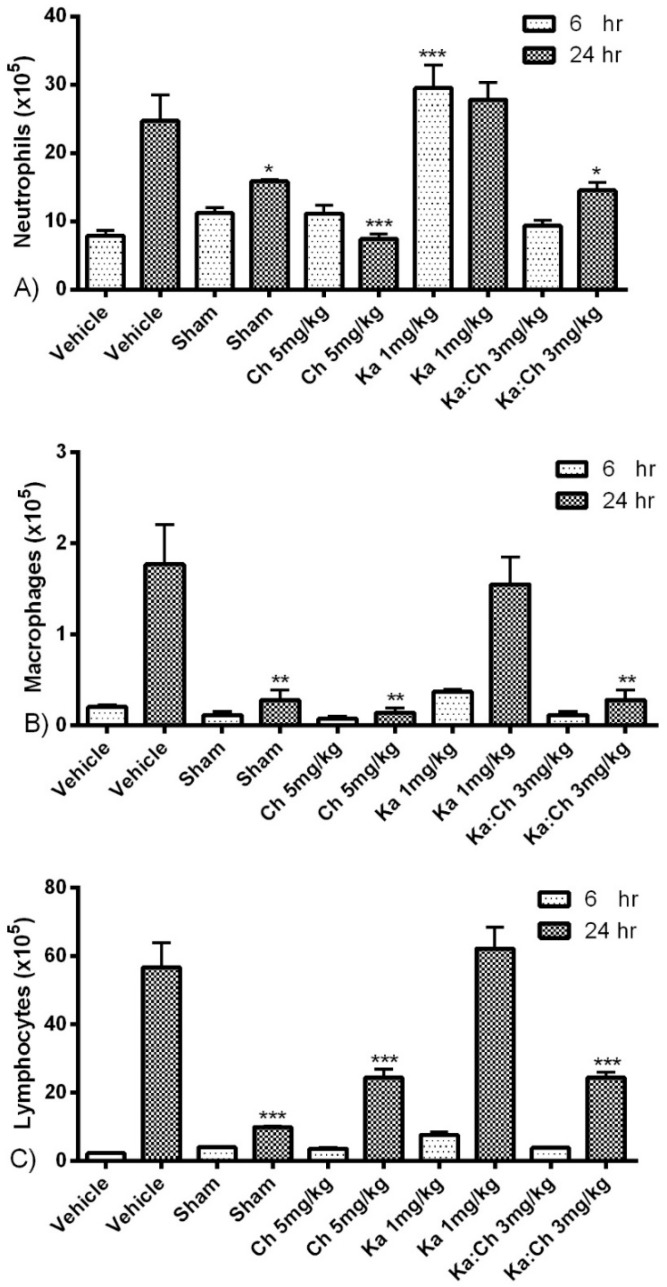
The effects of kaempferol and chrysin individual and combination treatments on IPF differential leukocytic count 6 and 24 h after CLP; (**A**) neutrophils; (**B**) macrophages; and (**C**) lymphocyte. Cells of lavaged IPF were fixed on slides through cytospin and then Wright’s stained for differential leukocytic count. Data represent actual cell numbers. * *p* ≤ 0.05, ** *p* ≤ 0.01, and *** *p* ≤ 0.001 versus CLP/vehicle of the same time point (Student’s *t*-test).

Results from Figure 4 show an increasing trend in total leukocyte cell population from 6 to 24 h. Kaempferol at 1 mg/kg significantly increased the leukocytes numbers at 6 h (*p* ≤ 0.001) and maintained this high population for up to 24 h. Meanwhile, chrysin at 5 mg/kg and the combination of kaempferol/chrysin at 3 mg/kg maintained a significantly low leukocyte population at 24 h compared to the vehicle control group. Likewise, the kaempferol treatment at 1 mg/kg maintained high neutrophil, macrophage, and lymphocyte populations, as displayed in Figure 5, while the chrysin (5 mg/kg) and kaempferol/chrysin (3 mg/kg) treatments maintained significantly lower populations compared to the vehicle control group.

### 2.4. Bacterial Clearance

**Figure 6:**
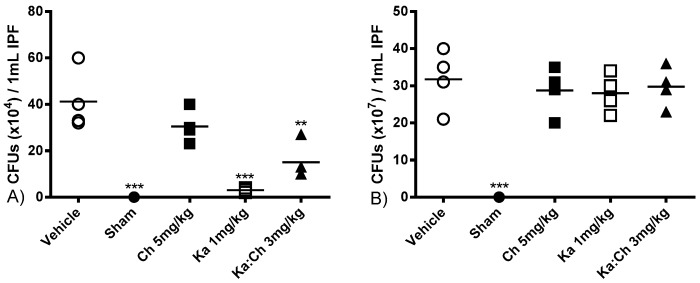
The effects of kaempferol and chrysin individual and combination treatments on bacterial colony forming units (CFUs) growth number/mL of IPF, at (**A**) 6 and (**B**) 24 h post-CLP. Abdominal swabs plated on nutrient agar and incubated for 24 h prior to CFU count. Data are presented as mean CFUs/mL. ** *p* ≤ 0.01 and *** *p* ≤ 0.001 versus CLP/vehicle of the same time point (Student’s *t*-test).

The antibacterial properties of kaempferol and chrysin individual or combination treatments were assessed by counting the colony forming units (CFUs) at 6 and 24 h post-CLP followed by a 24 h incubation period in nutrient agar. As shown in Figure 6, intraperitoneal swabs from the sham-operated group were sterile at 6 and 24 h. Even though the kaempferol (1 mg/kg) and kaempferol/chrysin (3 mg/kg) combination groups significantly decreased the CFUs at 6 h with *p* ≤ 0.001 and *p* ≤ 0.01, respectively, the chrysin (5 mg/kg) group did not significantly attenuate the CFUs at 6 h, and none of the tested groups exhibited antibacterial effects at 24 h.

### 2.5. Serum AST and TNF-α Levels

**Figure 7:**
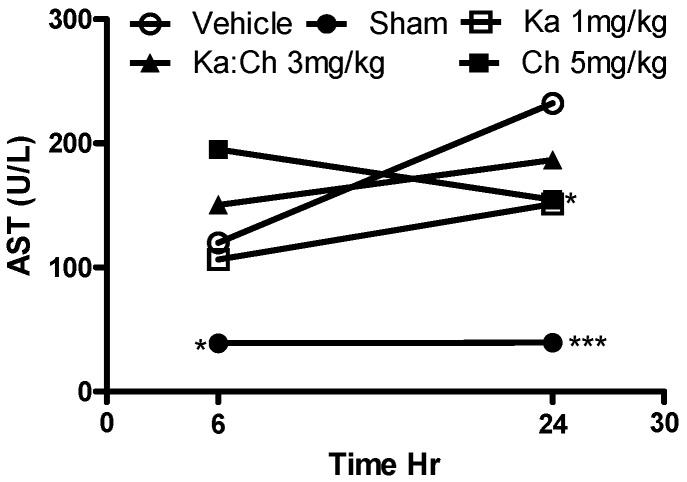
Time course profile of kaempferol and chrysin individual and combination treatments on serum aspartate aminotransferase (AST) levels in septic mice determined using ELISA kit. Each point represents the mean of four experiments. * *p* ≤ 0.05 and *** *p* ≤ 0.001 versus CLP/vehicle (Student’s *t*-test).

**Figure 8:**
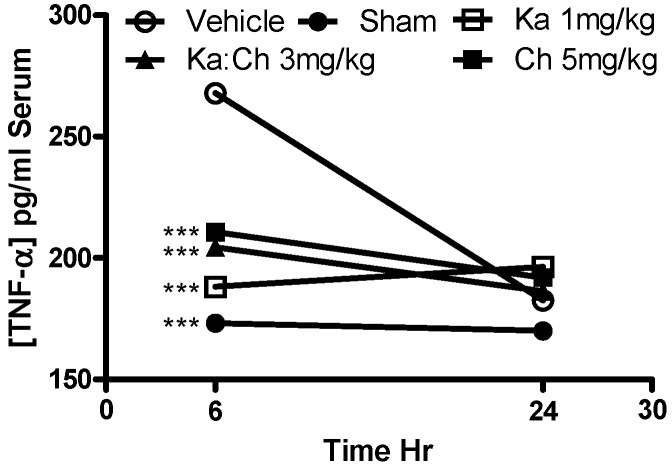
Time course profile of kaempferol and chrysin individual and combination treatments on serum tumor necrosis factor-alpha (TNF-α) levels in septic mice determined using ELISA kit. Each point represents the mean of four experiments. *** *p* ≤ 0.001 versus CLP/vehicle (Student’s *t*-test).

The serum fibrosis marker, AST, was measured as an indicator to assess the degree of tissue damage, particularly heart and liver inflammation. Figure 7 shows an overall increase in AST levels in most of the treatment groups during the progression of sepsis from 6 to 24 h. Kaempferol at 1 mg/kg showed a significant decrease in AST levels (*p* ≤ 0.05) compared to the CLP/vehicle control group at 24 h, while the sham-operated group demonstrated a baseline AST level of 39 U/L. As shown in Figure 8, the serum TNF-α levels decreased during the progression of sepsis from 6 to 24 h, and all treatments showed a significant reduction in serum TNF-α levels (*p* ≤ 0.001) at 6 h compared to the CLP/vehicle group.

### 2.6. Nitrite Determination

**Figure 9:**
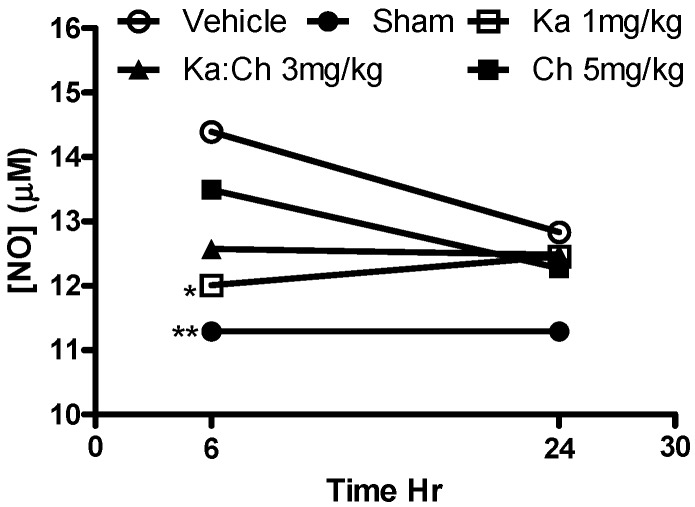
Time course profile of kaempferol and chrysin individual and combination treatments on serum nitrite levels in septic mice determined fluorometrically using 2,3-diaminonaphthalene (DAN). Each point represents the mean of four experiments. * *p* ≤ 0.05 and ** *p* ≤ 0.01 versus CLP/vehicle (Student’s *t*-test).

Figure 9 shows that only kaempferol at 1 mg/kg significantly reduced serum nitrate levels (*p* ≤ 0.05) at 6 h compared to the CLP/vehicle control group. Meanwhile, the sham-operated group maintained a baseline nitrite level.

### 2.7. MPO Activity in Liver Tissue

MPO activity in liver tissue was measured as an index to assess the extent of liver injury resulting from neutrophil infiltration during the progression of sepsis. Tissue MPO activities reached a peak at 6 h after CLP operation, followed by a general decrease at 24 h. All treatment groups showed a significant reduction in MPO activities at 6 and 24 h compared to the CLP/vehicle. Out of all the groups, chrysin at 5 mg/kg and kaempferol/chrysin 3 mg/kg combination groups reduced MPO activity to nearly that of the sham-operated control group level (Table 1).

## 3. Statistical Analysis

Statistical analysis was performed using GraphPad Prism version 5.00 for Windows (GraphPad Software, San Diego, CA, USA, http://www.graphpad.com). Survival rates were expressed as percentages, and differences between groups were analyzed using the Kaplan–Meier log-rank and chi-square test. The data were presented as mean ± standard error of the mean (SEM) and compared using Student’s *t*-test.

## 4. Materials and Methods

### 4.1. Animals

Male ICR mice (25–30 g) were used in this experiment. The mice were maintained in a temperature- and humidity-controlled room with a 12 h light/dark cycle and allowed free access to standard chow diet and water. The experimental protocol was approved by the Animal Care and Use Committee of the Faculty of Medicine and Health Sciences, Universiti Putra Malaysia (Approval no. UPM/FPSK/PADS/BR-UUH/00309). Chrysin and kaempferol were purchased from Sigma-Aldrich (St. Louis, MO, USA). All compounds were dissolved in 2% dimethylsulfoxide (DMSO) and 5% Tween-20/phosphate-buffered saline (PBS).

### 4.2. Experimental Protocol and Combination

The mice were randomly stratified and divided into 15 groups (*n* = 7) including, briefly, the sham-operated group, CLP-vehicle treated group (2% DMSO and 5% Tween-20/PBS), CLP-chrysin-treated groups (with 50, 10, 5, 1, and 0.1 mg/kg), CLP-kaempferol-treated groups (with 50, 10, 5, 1, and 0.1 mg/kg), and CLP-kaempferol/chrysin combination treatment groups (with 6, 3, and 1.5 mg/kg). All mice in this study received the same volume (10 mL/kg) in a single injection 1 h prior to the CLP procedure. Combination doses were determined based on the optimum survival dose of each single compound. A fixed-ratio proportion was selected by combining the best survival dose of each single compound followed by a 2-fold serial dilution.

### 4.3. CLP as a Sepsis Model

Mice were acclimatized for 7 days, then starved for 12 h with free access to water to empty their bowels. Sepsis was induced in all groups of mice via the CLP procedure according to the method reported by Rittirsch et al. [29]. Briefly, mice were anesthetized with 2,2,2-tribromoethanol (St. Louis, MO, USA, 250 mg/kg i.p.) [30], and unconsciousness was tested via animal paw pinching. After shaving the lower quadrants, a 1 cm midline incision at the linea alba was made under sterile conditions, the cecum was then exposed and ligated immediately–distal to the ileocecal valve—so that the bowel continuity would be preserved. A single “through and through” perforation with an 18-gauge needle was made through the distal cecum, after which the fecal contents were extruded into the peritoneum. After the puncture, the cecum was relocated into the abdominal cavity, and the peritoneal incision was closed in layers with simple running sutures (*BRILON*, *VIGILENZ non-absorbable*, Malaysia). The sham control animals were subjected to midline laparotomy, in which their cecum was manipulated but neither ligated nor punctured. The mice were allowed to recover in their cages postoperatively with free access to rodent chow and water, and antibiotic cream was applied once at point of surgery.

### 4.4. Survival Rates, Harvesting Samples, and Cytokine Determination

Survival was monitored up to 7 days after the operation. In an additional set of experiments, the mice from each group were sacrificed at two time points of 6 and 24 h after operation for sample harvesting. Blood was collected via cardiac puncture, and then centrifuged and stored at −20 °C until the serum levels of AST and TNF-α were determined using commercially available ELISA kits (ID Labs Inc., London, ON, Canada, Cat No. SUP6002 and BD Pharmingen, San Diego, CA, USA, Cat No. 555268, respectively). Thymus and spleen were rapidly harvested and weighed, and liver tissues were harvested for MPO activity measurement. The peritoneal cavity was lavaged with 3 mL of sterile PBS and the obtained intraperitoneal fluid (IPF) was kept on ice for further analysis. IPF (500 µL) was fixed on slides by cytospin at 500 RCF (relative centrifugal force) for 5 min and stained with Wright’s stain to determine the differential leukocyte count (1 slide/mouse, 4 mice/group, 300 cells counted/slide); 10 μL of IPF was stained with trypan blue for polymorphonuclear leukocyte quantification using a hemocytometer under a light microscope; whereas 100 μL of IPF was processed and serially diluted under sterile conditions on nutrient agar plates (Merck, Darmstadt, Germany) for bacterial-forming colony quantification. The same investigator carried out the standardized operations, sample collection, and processing of this study.

### 4.5. Serum Nitrite Determination

The serum nitrite concentration was determined spectrofluorometrically, as described by Fernández et al. [17]. Nitrite determination was based on its ability to react with 2,3-diaminonaphthalene (DAN) under acidic conditions to yield the fluorescent product 1-(*H*)-naphthotriazole. Briefly, 10 μL of freshly prepared DAN (0.05 mg/mL in 0.62 M HCl) was added to 50 μL of either the samples or nitrite standards in wells and then mixed. After 10 min incubation at room temperature in the dark, the reaction was stopped by adding 5 μL of 0.28 N NaOH per well. The formed 1-(*H*)-naphthotriazole was measured fluorometrically with an excitation wavelength of 350 nm, and the emission was read at 450 nm in a microplate spectrofluorometer (TECAN, Infinite M200, Männedorf, Switzerland). The concentration of nitrite in the samples was calculated from a sodium nitrite standard curve (0–100 μmol/L). The samples were performed in quadruplicates.

### 4.6. Measurement of MPO Activity in Liver Tissue

MPO was measured in liver tissues, as described by Hillefass et al. [31]. Briefly, 100 mg of liver tissue was homogenized and suspended in 50 mM of potassium phosphate buffer (pH 6.0) on ice. Then, it was centrifuged at 20,000× *g* at 4 °C for 15 min. The supernatant was discarded and pellet was resuspended in 50 mM of phosphate buffer containing 0.5% hexadecyltrimethyl-ammonium bromide (HTA-Br) (St. Louis, MO, USA). The tube contents were then homogenized for 30 s and sonicated with an ultrasonic dismembrator (Branson, Danbury, CT, USA) for 15 s at 40% power. Three cycles of freezing and thawing in liquid nitrogen were performed, followed by repeated sonication. The tube was centrifuged again at 20,000× *g* at 4 °C for 15 min and the supernatant aliquots (100 μL) were added to 2.9 mL of the reaction mixture, which contained 50 mM of potassium phosphate buffer, 0.167 mg/mL *o*-dianisidine dihydrochloride, and a 0.0005% hydrogen peroxide (H_2_O_2_) solution. MPO activity in each sample was determined by measuring the changes in absorbance per 30 s interval over 3 min at 460 nm using a spectrophotometer (UVM 340, ASYS Hitech GmbH, Eugendorf, Austria). MPO activity was expressed as a percentage change in absorbance/100 mg liver tissue.

## 5. Discussion

Previous studies demonstrated that some flavonoids exhibited synergistic properties in vitro that were translated into an enhanced attenuation of the production of major proinflammatory mediators. Particularly, kaempferol and chrysin were reported to be highly synergistic in inhibiting the production of NO, TNF-α, and PGE_2_ from LPS-induced RAW 264.7 murine macrophages by reducing the IC_50_ (inhibitory concentration at 50%) of the individual compound [14]. In view of this finding, this study aims to investigate whether or not the synergistic effects previously achieved in vitro could be translated into an animal model of sepsis.

During sepsis progression, the vehicle-treated control group died rapidly within the first 3 days. A postmortem necropsy examination showed that septic mice died due to severe multiple organ dysfunction and necrosis, which were reflected by the reduction of the thymus and spleen mean weights during the progression of sepsis. On the other hand, the sham-operated group survived until the end of the experiment, and the organs of this group did not undergo any weight reduction, as they were not challenged by polymicrobial sepsis. Based on the results of our present study, kaempferol and chrysin did not synergize in inhibiting many of the proinflammatory markers, but instead exhibited different patterns of protection against multiple inflammatory factors in the septic mice. However, their combination significantly improved the outcome of sepsis as a whole, described by the increase in the survival rate of the septic animals of up to 29%, which is considered a significant improvement compared to the individual kaempferol/chrysin treatments. Therefore, this finding suggests that kaempferol/chrysin combination may possess underlying synergistic benefits.

Kaempferol or chrysin and their combination expressed an additive protective effect against the serum levels of AST, NO, and TNF-α in septic mice. Interestingly, it was noted that kaempferol or chrysin treatments and their combination significantly attenuated serum TNF-α and NO levels when these cytokines were majorly expressed in the septic animal at the early phase of sepsis, followed by a general depletion of their concentrations in all groups at a later phase, which is attributed to the short lifespan of these cytokines. These findings align with previously reported beneficial anti-inflammatory activities of kaempferol and chrysin through the inhibition of the overproduction of TNF-α and NO [32,33]. Accordingly, the abilities of kaempferol and chrysin to attenuate these cytokines as key targets in septic animals have contributed to the initial survival benefits in response to the CLP-induced lethal sepsis in this model.

Sepsis is characterized by the early systemic recruitment of PMNs, which are short-lived and rapidly undergo apoptosis [34], followed by the recruitment of monocytes and lymphocytes, accordingly [35]. White blood cells (WBCs), neutrophils, monocytes, and macrophages have been reported to be highly recruited and activated following the release of inflammatory mediators such as TNF-α and ·NO in the animal system during the progression of sepsis [36]. Similarly, this study demonstrated rising trends in total WBC count and the percentages of cells also shifted from neutrophils towards lymphocytes during the progression of sepsis. Interestingly, the individual treatment of kaempferol further promoted WBC recruitment, pertaining to a significant increase in total WBC count by 2-fold at 6 h post-CLP; subsequently, a high WBC count similar to that observed in the vehicle-treated group at 24 h was maintained. On the other hand, chrysin treatment was found to attenuate WBC count to the basal levels of the sham-operated group. As expected, the kaempferol/chrysin combination treatment had an additive intermediate effect upon the WBC population when compared to that of individual treatments. This unique ability of kaempferol to increase neutrophil population could be attributed to its previously reported distinctive antimicrobial and antibacterial properties [37,38]. Interestingly, even though kaempferol treatment attenuated bacterial colonies at the early stages of sepsis, it failed to sustain a prolonged antibacterial effect. This suggests that any prolonged therapeutic effects by kaempferol is attributed to its anti-inflammatory and antioxidant activities alongside its short-term antibacterial activities. Moreover, kaempferol’s ability to sustain a high neutrophil count at 24 h indicates that kaempferol may possess some antiapoptotic activities, which is supported by its ability to reduce the serum fibrosis marker (AST) levels at the same time point. Such properties of kaempferol have been previously reported during inflammation [39].

Neutrophil activation and migration to the site of infection is believed to play a complex protective role during sepsis and inflammation. Activated neutrophils release a host of mediators, cytokines, free radicals, MPO, and phagocyte bacteria before apoptosis [36]. During an impaired immune response, delayed neutrophil apoptosis has been associated with high serum levels of activated neutrophils and reported to cause tissue injury and SIRS during sepsis [40,41]. Furthermore, the neutrophil-secreted MPO enzyme has been linked to oxidative stress and tissue damage [42], and is used as an independent prognostic marker for neutrophil activation and migration [43]. Likewise, this study reported a significant increase in liver tissue MPO activities in CLP-challenged groups. However, chrysin treatment attenuated MPO activities to almost basal levels, whereas kaempferol treatment did not exhibit similar benefits. Accordingly, the kaempferol/chrysin combination expressed an intermediate additive effect upon MPO inhibition compared to that of individual treatment.

In conclusion, this study demonstrated that a combined treatment of kaempferol and chrysin significantly improved the outcome of CLP-induced septic shock. This combination may not have a superior effect on any pathophysiological factor compared to the individual treatments, for which each flavonoid acted on different factors independently. However, when combined, kaempferol and chrysin synergized and significantly increased the survival of septic animals. These findings suggest kaempferol/chrysin combinational treatment as a beneficial adjuvant therapy in sepsis control, opening further possibilities of research to investigate the potentials and mechanisms of different flavonoid combinations, synergism, and protection against sepsis.

## Figures and Tables

**Table 1:** The effects of kaempferol (Ka) and chrysin (Ch) individual and combination treatments on liver tissue myeloperoxidase (MPO) activity.

Absorbance Change/100 mg Liver Tissue ×100				
	6 h		24 h	
Vehicle	311 ± 40		266 ± 40	
Sham	111 ± 30	***	78 ± 40	***
Ch 5 mg/kg	134 ± 30	***	82 ± 20	***
Ka 1 mg/kg	221 ± 20	**	167 ± 60	**
Ka:Ch 3 mg/kg	140 ± 10	***	99 ± 20	***

Data presented as change of absorbance/100 mg liver tissue, each value is the mean ± SEM of four experiments. ** *p* = 0.01 and *** *p* = 0.001 versus CLP/vehicle of the same time point (Student’s *t*-test).
